# Developing a disease-specific accessible transcriptional signature as a biomarker for ataxia with oculomotor apraxia type 2

**DOI:** 10.1186/s10020-025-01257-8

**Published:** 2025-05-24

**Authors:** Kathie J. Ngo, Darice Y. Wong, Alden Y. Huang, Hane Lee, Stanley F. Nelson, Brent L. Fogel

**Affiliations:** 1 Department of Neurology, David Geffen School of Medicine, University of California Los Angeles, Los Angeles, CA USA; 2 Clinical Neurogenomics Research Center, David Geffen School of Medicine, University of California Los Angeles, Los Angeles, CA USA; 3 Institute for Precision Health, David Geffen School of Medicine, University of California Los Angeles, Los Angeles, CA USA; 4 3billion, Inc., Seoul, Korea; 5 Department of Pathology and Laboratory Medicine, David Geffen School of Medicine, University of California Los Angeles, Los Angeles, CA USA; 6 Department of Human Genetics, David Geffen School of Medicine, University of California Los Angeles, Los Angeles, CA USA

**Keywords:** Biomarker, Senataxin, AOA2, Ataxia with oculomotor apraxia type 2, RNA-sequencing, WGCNA

## Abstract

**Background:**

Genetic ataxias are clinically heterogenous neurodegenerative conditions often involving rare or private mutations and it is often difficult to assign pathogenicity to rare gene variants solely based on DNA sequencing. An effective functional assay from an easy-to-obtain biospecimen would aid this assessment and be of high clinical value. *SETX* encodes a ubiquitous DNA/RNA helicase crucial for resolving R-loops and maintaining genome stability. Loss-of-function mutations cause a recessive disorder, Ataxia with Oculomotor Apraxia Type 2 (AOA2).

**Methods:**

Here we utilize Weighted Gene Co-expression Network Analysis (WGCNA) from patient blood to construct an AOA2-specific transcriptomic signature as a biomarker to evaluate *SETX* variants in patients clinically suspected of having AOA2.

**Results:**

WGCNA from peripheral blood RNA of 11 AOA2 patients from 7 families initially identified a single gene module that was modestly effective in distinguishing individuals with AOA2 from controls (sensitivity 73%, specificity 97%) and was able to robustly differentiate AOA2 patients from those with genetically distinct, yet phenotypically similar, neurological disorders (sensitivity 100%, specificity 100%). An independent derivation of the transcriptional biomarker identified a dual module model that was able to better distinguish individuals with AOA2 from controls (sensitivity 100%, specificity 97%). As validation, we examined a second cohort of 21 patients from 13 families and demonstrate that this dual module transcriptional biomarker could discriminate patients clinically suspected of AOA2 from controls (57%, 95%CI: 34%—78%).

Overall, the transcriptional biomarker was able to separate AOA2 subjects (*n* = 32) from controls (*n* = 35) with 72% sensitivity and 97% specificity. Notably, this transcriptomic biomarker enabled verification of the first pathogenic *SETX* mutation found in a non-canonical transcript, expanding the spectrum of mutations that contribute to AOA2.

**Conclusions:**

Our study identified a transcriptional biomarker that was able to differentiate AOA2 from controls and from other related neurological disorders, consequently expanding the spectrum of known pathogenic mutations. This proof-of-concept study illustrates that transcriptional biomarkers may be used to validate variants of uncertain significance in known genetic diseases.

**Supplementary Information:**

The online version contains supplementary material available at 10.1186/s10020-025-01257-8.

## Introduction

Molecular diagnosis of rare genetic neurological diseases poses a significant challenge, specifically in assessing the pathogenicity of variants through DNA sequencing alone. Variants of uncertain clinical significance (VUS) within such genes present challenges in characterization. Rare private gene variations, exclusive to a limited number of individuals or confined to specific families, add another layer of complexity to variant analysis and interpretation. Traditional methods to study gene function, such as using in-vitro assays or creating variant-specific animal models, are not always available, feasible, or financially practical and are often time and resource consuming. Adding further complication, distinguishing diseases that have shared phenotypes becomes challenging in the absence of specific clinical or biochemical features, as shared clinical presentations can impede precise differential diagnosis. Having an easily accessible disease-specific biomarker could not only resolve these issues, but also provide clinical utility for testing the efficacy of potential therapeutic agents. Transcriptional analysis to establish disease-specific profiles or signatures has been suggested as a means to develop clinically useful biomarkers (Riedmaier and Pfaffl 2013; Haq et al. 2025; Zhu et al. 2025). To address these challenges, we set out to use transcriptional profiling as an innovative approach for biomarker discovery in neurodegenerative disease. Identifying gene expression signatures that correlate with specific diseases could be valuable for developing a diagnostic or prognostic biomarker to monitor disease severity and/or progression. This strategy not only reduces reliance on the resource-intensive nature of traditional functional studies but could offer a more efficient means of elucidating functional implications of genetic variants in disease context with the potential for tracking disease status in real time.

Ataxia with Oculomotor Apraxia Type 2 (AOA2) presents as a progressive neurodegenerative disorder with adolescent-onset cerebellar ataxia, oculomotor apraxia in at least 50% of patients, elevated serum alpha-fetoprotein (AFP) levels, and peripheral sensorimotor axonal neuropathy leading to patients ultimately becoming unable to care for themselves (Anheim et al. 2009; Bennett and La Spada 2018; Fogel et al. 2014). The complexity in diagnosing AOA2 is amplified by its phenotypic similarities to other ataxic disorders featuring oculomotor apraxia, including ataxia oculomotor types 1 and 4 (AOA1, AOA4), XRCC1 (spinocerebellar ataxia, autosomal recessive 26; SCAR26), ataxia-telangiectasia (A-T), and ataxia-telangiectasia-like disorder 1 and 2 (ATLD1, ATLD2). In addition, AOA2 also has shared features with the most common recessive form of cerebellar ataxia, Friedreich ataxia (FRDA) (Fogel 2012; Fogel and Perlman 2007) as well as other rare ataxic disorders. These diagnostic challenges are further compounded by the continuous emergence of private variants in the *SETX* gene since the protein is quite large, at 2,677 amino acids, and consequently the locus is quite polymorphic. These challenges involved in diagnosing patients with AOA2 make it an ideal candidate for utilizing transcriptional profiling as a proof-of-concept study.

Previously, our group has used weighted gene co-expression network analysis (WGCNA) to identify transcriptomic networks from peripheral blood of AOA2 patients and controls, suggesting the possibility such networks could be used as a disease-specific biomarker (Fogel et al. 2014). Using microarray analysis, WGCNA identified two modules, turquoise and blue, that were AOA2 disease-specific. The turquoise module was characterized to be a *SETX* functional module while the blue module was characterized to be an AOA2 disease-specific module. Both modules were preserved in AOA2 patient cultured fibroblast tissues as well as in the cerebellum of a *Setx*^−/−^ knockout mouse model suggesting that the expression profile was conserved across tissues and species (Fogel et al. 2014). *Setx*^−/−^ knockout mice are not ataxic and do not have a neurological phenotype. Instead, they have a reproductive phenotype where male mice are sterile. While originally overlooked as unrelated to human disease, the close comparison of the turquoise module from patient blood to the disease associated greenblue module from knockout mouse cerebellum subsequently led to the identification of a novel phenotype of male infertility in AOA2 patients (Becherel et al. 2019), not previously recognized as a clinical feature of AOA2. This result supported the diagnostic relationship between the identified transcriptional signature and disease as it predicted a novel phenotype that was not previously associated with AOA2 (Becherel et al. 2019). In a similar study, our group used WGCNA to identify transcriptomic networks from peripheral blood of controls and ALS4 (Amyotrophic Lateral Sclerosis Type 4) patients, a motor neuron disorder also caused by mutations in the *SETX* gene (Hadjinicolaou et al. 2021). Comparisons of the transcriptomic networks from both studies supported transcriptional profiling in identifying disease-specific molecular signatures between AOA2 and ALS4 (Hadjinicolaou et al. 2021). Although clinically informative, both studies lacked sufficient statistical power to further extend the transcription profiling to a biomarker applicable to all AOA2 patients (Fogel et al. 2014) or all ALS4 patients (Hadjinicolaou et al. 2021) due to limited patient numbers, low sensitivity, and limited resolution of the transcriptional data. In this investigation, we sought to develop a more robust disease biomarker utilizing RNA-sequencing in a larger population of AOA2 patients to establish expression profiles which could then be used to validate patients clinically suspected of having AOA2.

## Methods

### Study participants

Thirty-two study participants from 20 families were chosen based on a clinical phenotype matching AOA2, which encompasses information derived from neurological examinations, neuroimaging, electrophysiological studies, and AFP testing. Overall, 59% of the cohort was male (19/32) with an average age at onset of 15.5 years old (standard deviation 6.1 years, range 4–28 years) and primarily of European (72%) descent (Table 1, Supplemental Table S1). Patients had various types of clinical genetic testing including targeted *SETX* gene sequencing, gene panel testing, or genome-wide sequencing (whole exome or whole genome sequencing). Participants were stratified based on the following criteria: those with a phenotypic presentation aligning with AOA2, coupled with sensorimotor axonal neuropathy, elevated AFP, and clear genetics (homozygous or compound heterozygous known pathogenic or loss-of-function mutations), were categorized as “Clinically Definite” (*n* = 23 subjects across 14 families, Table 1, Supplemental Table S1). Participants displaying a phenotypic presentation consistent with AOA2 with sensorimotor axonal neuropathy and/or an elevated AFP, but with unclear genetics due to a either identification of one or more variants of uncertain significance or only a single pathogenic or loss-of-function variant, were classified as “Clinically Probable” (*n* = 9 subjects across 6 families). The twenty-three subjects that were considered as clinically definite were then randomly assigned by family to either a training set (*n* = 11 subjects) or replication set (*n* = 12 subjects). All nine subjects that were considered as clinically probable were assigned to the replication set (Table 1, Supplemental Table S1). An additional 35 subjects were enrolled as controls, with the majority (83%, 29/35) being unaffected family members and either confirmed or presumed AOA2 carriers.

**Table 1:** Characteristics of the 20 AOA2 families included in this investigation

Set	Family #	Label	Variant^a^ (mRNA)	Variant^b^ (Protein)	Patient#	Age	Biomarker(Family)	Biomarker (Individual)
Training	AOA2-1	D	c.5927 T > G c.5929 C > T	p.Leu1976 Arg p.Leu1977Phe	1	16	x,o	x,o
					2	18		o
					3	16		x,o
	AOA2-2	D	c.5927 T > G c.820 A > G	p.Leu1976 Arg p.Met274 Val	4	-	x,o	x,o
	AOA2-3	D	c.3575_3576 delAT c.6834_6839 delAACAAA	p.Asp1192 Alafs*5 p.Lys2278_Thr2279 del	5	-	x,o	x,o
	AOA2-4	D	c.6215_6216 delAG c.7309 C > G	p.Glu2072 Valfs*10 p.Leu2437 Val	6	4	x,o	x,o
	AOA2-5	D	c.6106G > A c.7151 delG	p.Gly2036 Arg p.Cys2384Leufs*25	7	14	o	o
	AOA2-6	D	exon20 deletion (homozygous)	exon20 deletion (homozygous)	8		x,o	x,o
					9	17		x,o
					10			o
	AOA2-7	D	c.5267 T > C deletion (arr[hg38] 9q34.13(132268952–132271826))	p.Phe1756Ser deletion (includes exons 24–25)	11	21	x,o	x,o
Replication	AOA2-8	P	**c.6536 T > G** **c.7287 + 204G > A (c.2092G > A)**^**c**^	**p.Ile2179Ser** **p.? (p.Ala698 Thr)**^**c**^	12	6	x,o	x,o
	AOA2-9	D	c.5927 T > G c.6654 + 1G > T	p.Leu1976 Arg p.Ala2183_Met2218 del	13		-	-
	AOA2-10	D	c.2015_2016 insA c.2724 delG	p.Asn672Lysfs*22 p.Lys909 Asnfs*9	14		-	-
	AOA2-11^d^	P	c.4679 dupA	p.Asn1560Lysfs*4	15	15	x,o	x,o
					16	19		x,o
	AOA2-12	D	c.2747_2748 insAT c.6689 T > C	p.Met917Leufs*2 p.Met2230 Thr	17	15	o	o
					18	20		-
	AOA2-13	D	c.1484 T > C c.7447G > A	p.Leu495Pro p.Gly2483 Arg	19	15	x	-
					20			x
					21	15		-
	AOA2-14	P	**c.1256 T > G** (homozygous)	**p.Met419 Arg** (homozygous)	22		o	o
					23			o
					24			o
	AOA2-15^d^	P	c.389–1703 A > C	p.?	25	28	x,o	x,o
	AOA2-16	D	c.677 C > G c.6208 + 1G > A c.6397-?_8034 +?del (del exons 19–26)	p.Ser226* p.? p.?	26		-	-
	AOA2-17	D	c.2332 C > T (homozygous)	p.Arg778* (homozygous)	27	20’s	x,o	o
					28	20’s		-
					29	20’s		x
	AOA2-18	P	**c.1427_1432 delATTTGC** **c.6815 T > C**	**p.His476_Leu477 del** **p.Val2272 Ala**	30	5	x,o	x,o
	AOA2-19	P	**c.3208_3209 delCT** **c.361 C > A**	p.Leu1070Phefs*4 **p.Pro121 Thr**	31		x,o	x,o
	AOA2-20	D	c.5308_5311 delGAGA c.6029 A > G	p.Glu1770Ilefs*15 p.Asn2010Ser	32	5	x,o	x,o

D = Clinically definite, P = Clinically Probable, Age = age at onset (years), x = Identified by Single-Module Model (R22) as AOA2, o = Identified by Dual-Module Model (ME20 and ME31) as AOA2

^a^Based on NCBI reference sequence NM_015046.7. Novel variations are bolded

^b^Based on NCBI reference sequence NP_055861.3. Novel variations are bolded

^c^Based on Ensembl reference sequence ENST00000436441.5. Novel variations are bolded

^d^A second *SETX* mutation has not yet been identified

*is standard HGVS genetic variation nomenclature representing translation termination codon

### Cohort genotyping by targeted resequencing

DNA was isolated from each participant’s blood by standard methods. All participants were genotyped by a TruSeq custom amplicon panel that covered all the coding regions of the *SETX* gene excluding UTRs (Illumina Inc., San Diego, CA) to confirm variants and segregation analysis.

### Variant assessment

Variant rarity was assessed by comparing frequency to the publicly available databases gnomAD v4 (accessed March 4, 2024) and All of Us (https://databrowser.researchallofus.org/variants, accessed March 4, 2024) (Karczewski et al. 2020). Single nucleotide variants (SNVs) and indels were assessed for pathogenicity based on their ClinVar and HGMD (Human Gene Mutation Database) classification, CADD (Combined Annotation Dependent Depletion) v1.7 phred score prediction, and Alpha Missense classification if applicable (Cheng et al. 2023; Landrum et al. 2014; Schubach et al. 2024; Stenson et al. 2014).

### Use of published datasets

Microarray datasets from AOA2 patient peripheral blood, AOA2 patient derived fibroblasts, and Setx knockout mouse cerebellum were obtained from our prior study (GSE61400) (Fogel et al. 2014). The AOA2 patient peripheral blood microarray dataset (15 patients from 12 families) shares subjects with the RNA-sequencing training set (5 patients from 3 families) and replication set (3 patients from 3 families) described above. RNA-sequencing data from the peripheral blood of patients presenting with Phelan-McDermid syndrome (PMS) were obtained through the Sequence Read Archive (SRA) accession number SRP394132 (Breen et al. 2023). Following the processing method described below, one outlier sample was excluded from the analysis. RNA-sequencing data originating from Friedreich Ataxia (FRDA) patient fibroblasts were obtained using the SRA accession number SRP118922 (Napierala et al. 2017). Utilizing the same processing method, two outlier samples were excluded. Processed read count data from peripheral blood RNA-sequencing of ataxia-telangiectasia (A-T) patients were retrieved from the Gene Expression Omnibus (GEO) database using the accession number GSE142842 (McGrath-Morrow et al. 2020). WGCNA data from the peripheral blood of patients with Amyotrophic Lateral Sclerosis Type 4 (ALS4) were obtained from our prior study (Hadjinicolaou et al. 2021).

### Analysis of RNA-sequencing data

For this study, total RNA was extracted from peripheral blood samples of participants using an RNA library kit with RiboZero Gold treatment. Subsequently, sequencing was carried out on the HiSeq4000 platform, employing 75 bp paired-end reads (Illumina Inc., San Diego, CA). The sequence reads were then mapped to the GRCh38 human reference genome and Gencode GTF (v27) using the STAR spliced read aligner (v2.7.3a) (Dobin et al. 2013; Frankish et al. 2021). Picard tools (v2.25.1) were utilized for calculating Sequencing Q/C metrics (Broad Institute n.d.). The featureCounts tool (v2.0.1) was employed for read counting, and MultiQC (v1.10.1) generated summary sequencing metrics (Ewels et al. 2016; Liao et al. 2014). Samples showing low quality or DNA contamination were excluded at this stage. To ensure accuracy, a sex check was performed by examining *XIST* expression and Y chromosome non-pseudoautosomal expression, both known for distinct expression patterns between females and males respectively. A single gender mismatch was initially identified but was later confirmed to be a different family member who had been sequenced inadvertently. Furthermore, BAM files underwent scrutiny for *SETX* variations to ensure the absence of any sample swapping.

All datasets that had genes with zero counts were subsequently removed. The CQN (Conditional Quantile Normalization) package was used to correct for GC content, gene length, and quantile normalize the expression counts. The expression counts were then transformed to log_2_FPKM (Hansen et al. 2012). Linear regression was used to further clean up any artifacts from sex and sequencing batch effects if applicable with the SVA package v3.38 (Leek et al. 2012). Differential gene expression analysis was subsequently performed with DESeq2 package (Love et al. 2014).

### Weighted gene co-expression network analysis (WGCNA)

We employed Weighted Gene Co-expression Network Analysis (WGCNA) to construct a gene co-expression network using RNA-sequencing data extracted from participants’ peripheral blood. To minimize variability in gene expression due to technical artifacts and batch effects introduced by our analysis procedures, we analyzed the entire cohort simultaneously. WGCNA in this study was executed with the following parameters: a soft threshold power of 4, a minimum module size of 50, and a deep split of 2. Unique module labels (R01-24) were assigned to each module color, excluding the grey module as genes in this module are not co-expressed and not assigned to any module (Langfelder and Horvath 2008). Disease-associated modules were determined by correlation of the module eigengene with disease status across the subjects (unadjusted *p*-value < 0.05).

WGCNA was similarly conducted on three validation sets. Parameters for the soft threshold power (used for suppressing low correlations within each dataset) were chosen using the pickSoftThreshold function within the WGCNA package. For the PMS dataset, the network was generated using the parameters: a soft threshold power of 4, a minimum module size of 50, and a deep split of 2. The FRDA dataset’s network employed a soft threshold power of 8, a minimum module size of 50, and a deep split of 2. Finally, the A-T dataset’s network was created with a soft threshold power of 18, a minimum module size of 50, and a deep split of 2.

For comparison studies, we reanalyzed microarray datasets from our prior study (GSE61400), collapsing probes down to the gene level to align with the RNA-sequencing analysis mentioned earlier. In the patient peripheral blood dataset, WGCNA was performed with a soft threshold power of 3, a minimum module size of 50, and a deep split of 2. Unique module labels (B1-32) were assigned to each module color. For the patient-derived fibroblasts, WGCNA was conducted with a soft threshold power of 18, a minimum module size of 50, and a deep split of 2, and unique module labels (F1-86) were assigned. Similarly, in the mouse cerebellum dataset, WGCNA was conducted with a soft threshold power of 12, a minimum module size of 50, a deep split of 2, and a merge threshold of 0.1 (Fogel et al. 2014).

We utilized module preservation analysis to determine if the AOA2 disease-associated modules generated by WGCNA were generalizable to other validation datasets. Module preservation was carried out using the modulePreservation function within the WGCNA package (Langfelder et al. 2011). This analysis involved 50 permutation tests, and a randomSeed of 1 was utilized for consistency and reproducibility. We then used gene overlap analysis to assess if the module membership between datasets was significantly equivalent based on hypergeometric probability. We used the derived standard scores and probability values without further adjustment.

### Pigengene

The Pigengene v.1.29.10 R package was used as an independent alternative classifier method (Foroushani et al. 2017). Both the training set and replication set expression data was inputted to the one step pigengene function with following parameters: bnNum = 0, toCompact = FALSE, doHeat = TRUE to compute the classifier.

### Gene ontology enrichment analysis

Gene ontology (GO) analysis was performed using the DAVID Bioinformatic resources (Huang et al. 2009). Enriched GO terms with a *p*-value < 0.05 were subsequently inputted into the web tool REVIGO (reduce + visualize Gene Ontology) to reduce redundancy. All parameters were kept at default settings, except for the species parameter, which was set to *Homo sapiens* (Supek et al. 2011).

### Network visualization

Gene modules were imported into Cytoscape software (v3.9.1) from WGCNA to visualize the gene network (Saito et al. 2012). For module R22, the top 600 connections were selected for visualization. Undirected network analysis was performed, and the sizes of gene nodes were scaled based on the degree of connections for each specific node. Genes were organized and grouped according to highlighted GO categories.

### Statistical analysis

Differential gene expression was assessed with a threshold for statistical significance set at a false discovery rate (q) < 0.05. In Gene Ontology analyses, statistical significance was established with a *p*-value < 0.05. Enrichment statistical comparisons were conducted using hypergeometric probability, assuming *p* < 0.05 for determining significance. The comparison of module eigengene between the validation samples and controls was accomplished via a one-sample T-Test. Comparison between classifier methods (module R22 and two-module combination from Pigengene) was performed with Fisher’s Exact Test.

## Results

### Gene expression profiling of AOA2 patients from peripheral blood RNA-sequencing identifies gene modules associated with SETX function

#### Training Cohort

We sought to develop a disease-specific biomarker using transcriptional profiling in patients that were clinically verified as having AOA2. Since AOA2 is a recessive disorder commonly due to loss-of-function mutations, this transcriptional biomarker could also serve as a relative measure of SETX function. To test this, we used a training cohort of seven families (*n* = 11 patients, Table 1, Supplemental Table S1) with clinically definite AOA2, which included a subset of 11 patients with known pathogenic mutations based on American College of Medical Genetics and Genomics (ACMG) guidelines, along with 35 control subjects (Richards et al. 2015).

#### Weighted gene co-expression network analysis reveals AOA2 disease-associated networks are related to SETX function

We performed WGCNA on RNA-sequencing data generated from peripheral blood to identify AOA2 disease-associated transcriptional modules. WGCNA resulted in 24 modules that we designated as R modules (“RNA-sequencing”; Supplemental Table S2). Of the 24 modules, five modules were found to be AOA2 disease-associated: R05, R10, R20, R22, and R23. To determine if these five disease-associated modules are involved in biological processes and pathways pertaining to SETX function, we employed Gene Ontology (GO) analysis. GO analysis indicated that genes in modules R5 and R10 are involved in protein modification process, RNA metabolic process, and signal transduction biological processes (Supplemental Table S3-S4). GO analysis for module R20 suggest the genes in this module are involved in biological processes including DNA metabolic process, chromatin organization, and cell cycle processes (Supplemental Table S5). GO terms suggest that genes in module R22 are involved in biological processes including signal transduction, immune system development, DNA/RNA metabolic processes, and intracellular transport (Supplemental Table S6). Finally, GO terms for module R23 suggest the genes of this modules are involved in pathways resulting in breakdown of macromolecule/proteins, protein metabolic process, and protein modification process (Supplemental Table S7). These GO terms support that genes from these five modules are involved in biological processes or pathways pertaining to normal SETX function.

### Using AOA2 disease-associated modules to develop a transcriptional biomarker for AOA2

#### Transcriptional biomarker assessment in AOA2 training set

To determine if these AOA2 disease-associated modules could act as a biomarker and be used to classify patients as having AOA2, we leveraged the WGCNA module eigengene (ME) metric, defined as a representative of the gene expression profile within a module, as a classifier to determine the presence or absence of AOA2 (Langfelder and Horvath 2008). As AOA2 is caused by loss of gene function, this metric could also theoretically serve as an indirect proxy for SETX function. We utilized expression profiles from well assessed AOA2 patients assigned to the training group (*n* = 11 patients, Table 1, Supplemental Table S1) to establish a AOA2 disease expression profile for each of the five disease associated R modules. A one-sample T-test was then used to compare each individual patient’s expression profile to determine if the transcriptional profile was consistent with disease.

**Fig. 1:**
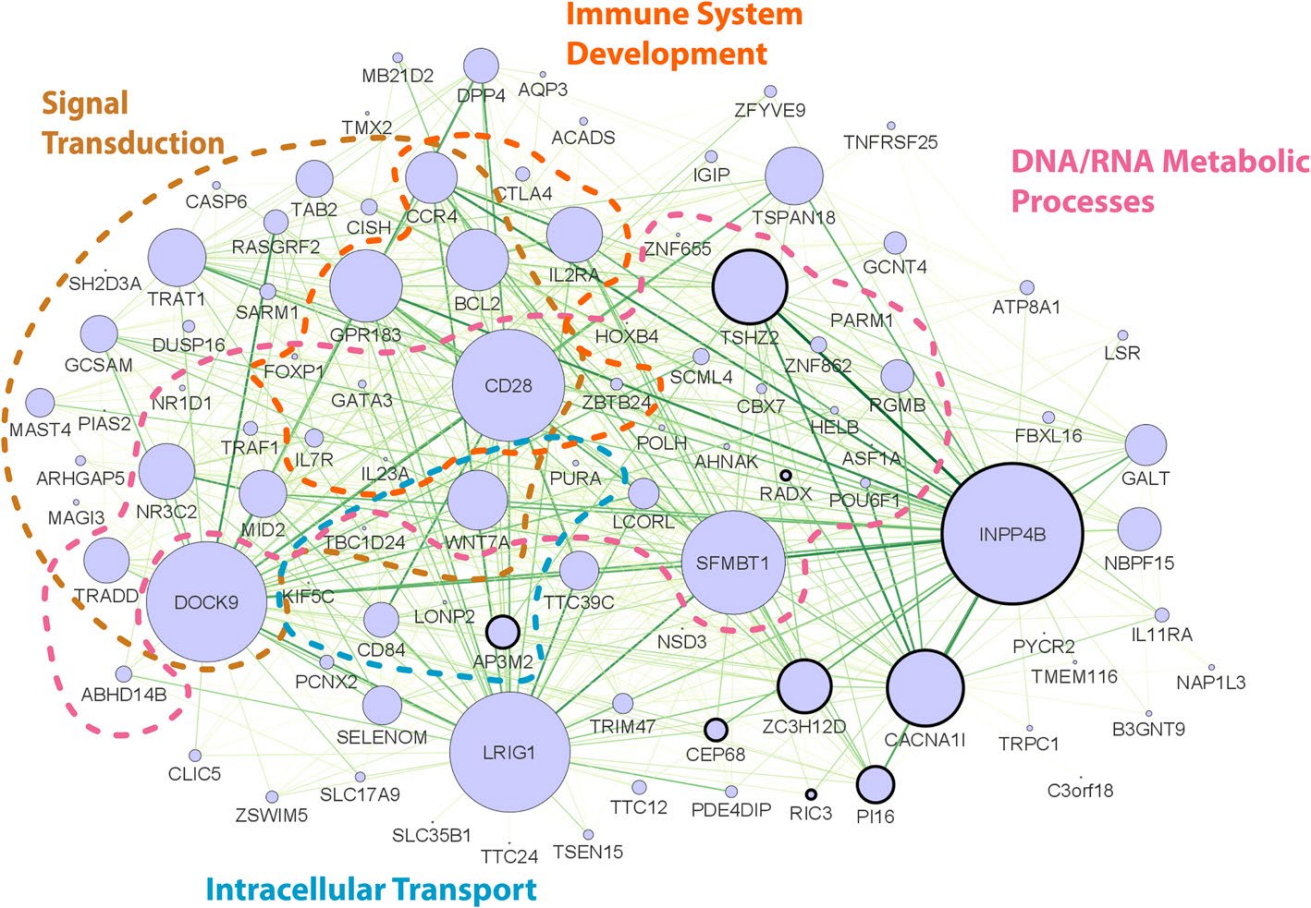
Module R22 Network Representation. Gene co-expression network from blood RNA-seq. Representation of the top interconnected genes in the R22 module. Top 600 connections are shown. Node size corresponds to the number of connections (degree). Hub genes (the most highly connected genes) are depicted as nodes of larger size. Nodes are organized into functional clusters based on significantly enriched gene ontologies. Genes that were found to be differentially expressed have a dark outline

We first examined which of the five modules, if any, were able to individually distinguish patients from the training set as AOA2 versus healthy control subjects. Of the five R modules, module R20 was the best single module to classify patient MEs as AOA2 with a sensitivity of 82% [95% CI: 48%—98%] whereas module R22 was the best single module to distinguish control subject MEs as healthy with a specificity of 97% [95% CI: 85%—100%] as well as having a high sensitivity of 73% [95% CI: 39% −94%], (Supplemental Table S8). Upon close inspection of the top 600 highly connected genes for module R22 (Fig. 1), we found that module R22 is enriched for a significant number of genes differentially expressed in AOA2 patients (10/25, *p* = 7.06E-17; Supplemental Table S9). Attempts to improve classification based on presence, absence, or magnitude of change in these differentially expressed genes did not improve classifier specificity or sensitivity (data not shown).

To see if we could further improve the sensitivity of the classifier, we next looked at combinations of modules to attempt to maximize the sensitivity and specificity. We tested all possible 2 module combinations and found that modules R10 and R22 was the best overall and maximized the sensitivity. Modules R10 and R22 in combination increased the sensitivity from 73% (R22 alone) to 100% [95% CI: 72%−100%] however the specificity decreased from 97% (R22 alone) to 80% [95% CI: 63%—92%] (Supplemental Table S8). We then attempted to improve the specificity by using three module combinations and found that R22 in combination with modules R10 and R20 yielded the maximum sensitivity of 100% [95% CI: 72%—100%] with a specificity of 91% [95%CI: 77%—98%] (Supplemental Table S8). In all cases, adding additional modules to R22 led to the identification of additional controls as positive (data not shown) and all further improvements to sensitivity were therefore deemed to result from bias due to overfitting of the training dataset. Consequently, to prioritize the accuracy of the classifier, this three-module combination (as well as the R10/R22 two-module combination) was subsequently rejected due to the associated risk of false positives. Combinations of more than three modules did not yield improved results. Therefore, we selected module R22 (sensitivity 73%, specificity 97%) for further analysis as a candidate transcriptional biomarker due to it having the best overall specificity and sensitivity combination.

#### Transcriptional biomarker cross-validation in an AOA2 replication set

**Fig. 2:**
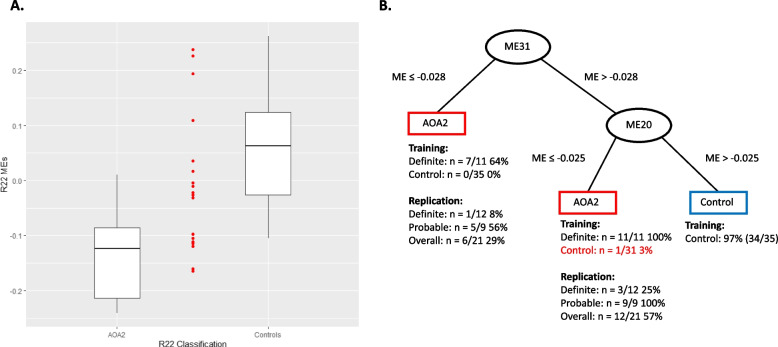
Biomarker classification of replication cohort. **A** Depiction of single module (R22) model classification of the patients from replication cohort (red dots) with respect to the distribution of the module eigengene (MEs) from the training cohort (box plots). **B** Depiction of the Two-Module Model classification of the patients from the replication cohort as a decision tree based on the MEs for each sample

To assess how well module R22 was able to classify AOA2 expression profiles outside of the training group, we utilized a replication group (Table 1, Supplemental Table S1) containing the remainder of the patients that were defined as clinically definite (*n* = 12) along with all patients that were defined as clinically probable (*n* = 9, Table 1, Supplemental Table S1). For this analysis, we considered families along with individuals and defined a family as positive if any of its affected members matched the module signature. We found that module R22, the best single module classifier with the highest specificity in the training group, classified 9 out of 21 patients (43%, 95%CI: 22%−66%; Fig. 2A). Module R22 further classified 8 out of 13 families as having AOA2 (62%, 95%CI: 32%−86%). No other modules, alone or in combination, proved superior to R22 alone (data not shown). This replication analysis supports module R22 as the most effective candidate transcriptional biomarker. In total, module R22 collectively identified 53% of all patients (17/32) and 70% of the families (14/20) between the training and replication cohorts (Table 1, Supplemental Table S1).

#### Independent derivation of the AOA2 biomarker

To independently assess our strategy for deriving the most effective transcriptional biomarker from our data, we utilized Pigengene, a decision tree modeling tool that can leverage the ME metric from WGCNA for classifying samples (Foroushani et al. 2017) assigned to our AOA2 training set. The WGCNA produced from the Pigengene tool resulted in three disease-associated modules: ME19, ME20, and ME31. We used gene overlap analysis to map and determine the most equivalent R modules that best matched the three ME modules. We found that module ME19 was the most equivalent to module R5 (*p* = 1.65E-123), module ME20 was the most equivalent to module R22 (*p* =1.23E-72), and module ME31 modestly overlapped with module R5 (*p* =1.48E-04). The remaining modules generated by the Pigengene tool also recapitulated the other modules found in our R modules showing concordance across the same dataset.

Next, we compared the results from the decision trees generated from the Pigengene tool to assess subject classification as having AOA2 or not. The Pigengene tool did not report any single module as a standalone classifier, so single module classification was not compared. When only ME20 (R22 equivalent) and ME31 (R5 equivalent) were used as a classifier, the decision tree was able to distinguish patients from controls with an improved sensitivity of 100% [95% CI: 72% −100%] and specificity of 97% [95% CI: 85%—100%] in the training cohort (Fig. 2B) versus module R22. When using all three ME modules in combination as a classifier, the specificity was maximized to 100% [95% CI: 90% −100%] but detailed results indicate this was again due to the overfitting, as seen with our previous method (data not shown).

Evaluation of the replication cohort with the two-module model of ME20 (R22 equivalent) and ME31 (R5 equivalent) yielded 57% (12/21, Fig. 2B) individuals and 69% (9/13) families classified as AOA2, only slightly improved versus module R22. In contrast, the two-module combination of R22 and R05 identified 81% (17/21) individuals and 85% (11/13) families as AOA2 in the replication cohort but had a high false positive rate with a specificity of 71% in the training set making it unsuitable as a biomarker. In summation, the two-module model developed using the Pigengene tool resulted in a sensitivity of 72% (23/32, [95% CI:53%−86%]) individuals (versus 53% (17/32, [95% CI: 35%−71%]) for the single-module model) and 80% (16/20, [95% CI: 56%−94%]) families (versus 70% (14/20, [95% CI: 46%−88%]) for single-module model) as AOA2 from the combined training and replication cohorts. We used a Fisher’s Exact Test to determine if the sensitivity from the two methods were statistically different. We found that the results from both methods were not significantly different when comparing either individuals (*p* = 0.196) or families (*p* = 0.716). Based on these cross-validation results, both methods resulted in overlapping modules that were able to successfully identify AOA2 patients and families. In conclusion, the two-module model derived from the Pigengene tool had the highest overall sensitivity and specificity in our cohort while module R22 was found to represent the most effective single-module module capable of differentiating AOA2 patients from controls with high specificity.

### Confirmation of module R22 as a key transcriptional biomarker for AOA2 across tissues and species

#### Biomarker confirmation in AOA2 patient blood, fibroblast, and Setx knock-out mouse models

To validate module R22 as the key single module AOA2 transcriptional biomarker and to assess concordance across tissues and species, we compared previous RNA expression datasets that include human peripheral blood, patient fibroblasts, and *Setx* knock-out (KO) mouse cerebellum from microarray (Fogel et al. 2014).

Our previous data had confirmed conservation of the AOA2 transcriptional signature across different tissues and species (Fogel et al. 2014). To compare the current biomarker to these previous datasets, we reanalyzed the WGCNAs to normalize the analysis (see Methods). The various re-analyses are referred to respectively as B modules (blood), F modules (fibroblasts), and M modules (*Setx* KO mouse cerebellum) respectively. For each dataset, we first characterized the new WGCNAs by looking for AOA2 disease associated modules and then used hypergeometric probability to confirm which of these were the equivalent modules for each of the previously published key modules (Fig. S1 A-C). After mapping the disease associated modules, we then compared these modules from blood (B02, B11, B14, B20, B26, B28), fibroblast (F08, F43, F56, F71, F79), and mouse cerebellum (M77) to our AOA2 disease-associated R modules (R05, R10, R20, R22, and R23) (Fig. S2). We observed corresponding relationships between the majority of all the key modules in the datasets with a notable correspondence between the modules identified in blood (R, B) and those identified in fibroblasts (F) (Fig. S2). Module R22, the best classifier module discussed above, only overlapped with module B14 (*p* = 4.20E-02) and did not have any significant overlaps with the other datasets (Fig. S2) suggesting that it may be blood-specific. In addition, to assess disease-specificity, we also included the key human blood module associated with ALS4 (ALS4HsModule77), a distinct dominant neuromuscular disorder associated with *SETX* but distinct from AOA2 (Fig. S2). Interestingly, module ALS4HsModule77 overlapped significantly only with module R05 (*p* = 8.16E-07, Fig. S2).

#### Confirmation of AOA2 transcriptional biomarker specificity versus other neurological disorders

To evaluate module R22 as a diagnostic AOA2 biomarker, we utilized the WGCNA module preservation function to discern whether module R22 could effectively distinguish broadly between the expression profiles of AOA2 and those of other neurodegenerative diseases. This investigation encompassed three distinct publicly available RNA-sequencing datasets as well as our prior ALS4 study (Hadjinicolaou et al. 2021). Among these datasets, two are phenocopies of AOA2, ataxia-telangiectasia (A-T) and Friedreich’s ataxia (FRDA)(McGrath-Morrow et al. 2020; Napierala et al. 2017), while the third dataset represented an unrelated neurodegenerative disorder, Phelan-McDermid syndrome (PMS) (Breen et al. 2023; Jesse et al. 2023). We chose this PMS dataset (a combination of patients with 22q13.33 microdeletion or *SHANK3* sequence variants) to evaluate specificity of the AOA2 transcriptional signature in a neurological disease with no phenotypic overlap. To assess the specificity of the AOA2 signature in diseases with phenotypic overlap we chose A-T and FRDA. Lastly, we evaluated disease specificity in comparison to ALS4, a phenotypically distinct disease caused by mutations within the same gene (*SETX*). We performed WGCNA and identified modules PMS9, PMS16, and PMS18 as disease-associated modules for PMS and module A-T13 as a disease-associated module in A-T. No disease-associated modules were identified in the FRDA dataset. Subsequently, module preservation analysis was conducted to assess the specificity of module R22 across all four datasets.

To determine whether the transcriptional profile was AOA2-specific, we defined the module R22 biomarker as positive if it was preserved in the other dataset and the most equivalent module was disease-associated. We found that module R22 was not preserved in either the A-T and FRDA datasets (z < 2) but was preserved in both the ALS4 (z = 2.07) and the PMS (z = 10.77) datasets (Fig. S3A). However, neither of the most equivalent R22 modules in these datasets (ALS4HsModule59 (*p* = 1.04E-03) and PMS5 (*p* = 9.50E-07) respectively) were disease-associated. These results confirm that the module R22 biomarker was not positive in any of these four other neurodegenerative disease datasets (Fig. S3A).

To provide additional certainty that the R22 biomarker is unique to AOA2, we sought to determine if any of the disease-associated expression profiles from these disease datasets were preserved in our AOA2 dataset and otherwise equivalent to the AOA2 signature (Fig. S3B). We considered these other disease modules to match AOA2 if any disease-associated modules were preserved and the most equivalent R module was module R22. We found that the A-T disease-associated module A-T13 was not preserved in our R modules (z < 2). There were 14 out of 33 modules that were preserved (z ≥ 2) from the FRDA dataset, but none were disease-associated. Both ALS4 disease associated modules: ALS4HsModule60 (z = 14.03) and ALS4HsModule77 (z = 7.49) were preserved in our R module dataset. From gene overlap analysis, we found that module R22 was not the most equivalent module for either ALS4 disease associated modules, although ALS4HsModule77 is most equivalent to module R5 (*p* = 8.16E-07) (Fig. S2), which is utilized in the best dual module model biomarker. Of the three PMS disease associated modules, only modules PMS9 (z = 9.87) and PMS18 (z = 10.42) were preserved in our R module dataset. Gene overlap analysis showed that module R22 did not have any significant overlap (*p* > 0.05) with the two preserved PMS disease associated modules. Together, this confirms that R22 is a unique transcriptional biomarker to our AOA2 dataset and not associated with these other four diseases evaluated (Fig. S3B).

### Use of the AOA2 transcriptional biomarker to validate a novel pathogenic mutation in a non-canonical alternatively spliced SETX transcript

**Fig. 3:**
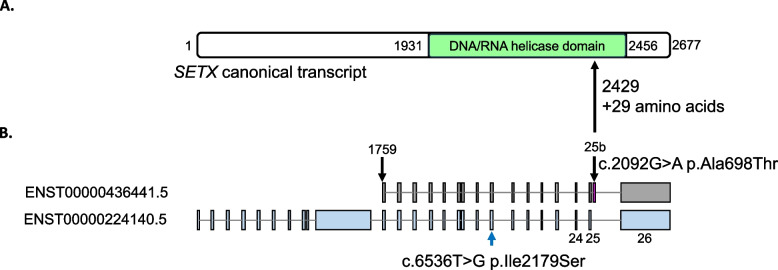
Novel pathogenic variant in an alternative isoform of *SETX*. **A** Depiction of the SETX protein showing the DNA/RNA helicase domain (green). **B** Depiction of SETX canonical transcript (ENST00000224140.5, bottom) and alternative isoform (ENST00000436441.5, top). Novel variant in the helicase domain indicated by blue arrow. Black arrows indicate the start of the shorter isoform with respect to the canonical isoform and the extra exon 25b (red) respectively

To test the efficacy of the AOA2 biomarker for validating novel *SETX* mutations, we examined patients clinically suspected of having AOA2, where genetic testing did not reveal two pathogenic or loss of function variants. In one specific case, we noted an instance where the AOA2 biomarker classified a patient as AOA2, but the patient only had one potentially diagnostic *SETX* variant on routine DNA testing. The patient was a 38-year-old woman who presented with early onset cerebellar ataxia. At age 6, she developed poor balance that began progressively worsening. By age 17, she presented with dysarthria along with gait, truncal, and limb ataxia. She had a mild peripheral sensorimotor neuropathy with weakness in all distal limbs as well as distal sensory loss and reduced deep tendon reflexes. MRI of the brain showed cerebellar atrophy. Laboratory studies included elevated alpha-fetoprotein (10.2 ng/mL; normal 0–7 ng/ml (Anheim et al. 2009)). AOA2 was clinically suspected but *SETX* gene sequencing yielded only a heterozygous missense variant of uncertain clinical significance (c.6536 T > G (p.Ile2179Ser); Table 1, Supplemental Table S1). The patient subsequently had whole exome sequencing with no other suspicious variants identified. Whole genome sequencing of the patient and her parents coupled with RNA-sequencing from blood identified another non-coding variant of uncertain significance in *SETX* c.7287 + 204 G > A (p.?). Segregation analysis showed that the noncoding variant was paternally inherited while the previously observed missense variant was maternally inherited. No additional suspicious variants were identified including copy number variation. Interestingly, RNA-sequencing showed both the patient and her father to have skewing of the alternate c.7287 + 204 G > A allele compared to controls subjects, being among the top expressers of this allele in blood (data not shown). We have previously shown that *SETX* is alternatively spliced (Fogel et al. 2009), but clinically and in the literature only the canonical transcript has previously been considered as disease-associated. An alternative *SETX* transcript was found (ENST00000436441.5) where the c.7287 + 204 G > A variant causes a missense change c.2092 G > A (p.Ala698Thr). This shorter isoform (948 amino acids) contains the entire SETX DNA/RNA helicase domain but with an extra exon, which we termed exon 25b, that adds an additional 29 amino acids after the corresponding position 2429 in exon 25 of the canonical *SETX* isoform (Fig. 3A and 3B). Our group has previously shown that exon 25b is expressed in both human fetal and adult brain and that adding exon 25b to the canonical transcript did not significantly alter its behavior compared to wildtype in patient cells (Fogel et al. 2009). GTEx isoform expression indicates this isoform is expressed in most brain regions to a greater extent than the canonical isoform suggesting this isoform may have a relevant role in SETX function (Lonsdale et al. 2013) within the brain. The patient’s transcriptional profile matched closely to that of the single-module R22 model (*p* = 1.77E-13) as well as the two-module model, consistent with an AOA2 expression profile. Conservatively, these results support the functional classification of the c.7287 + 204 G > A as likely pathogenic, per the ACMG guidelines (PS3, PM2, PM3, PP4)(Richards et al. 2015), and establish a genetic AOA2 diagnosis for this patient.

## Discussion

In this investigation we focused on developing a disease-specific transcriptional biomarker from accessible tissue for identifying patients with a neurodegenerative disease, specifically AOA2. To do so, we utilized the largest AOA2 cohort studied in this manner to identify an expression profile that could serve as a biomarker proxy for the absence of SETX function. Because SETX is ubiquitously expressed, we were able to leverage readily accessible tissues, such as whole blood, to generate a transcriptional profile from AOA2 patients that could be applied to validate patients clinically suspected of having AOA2.

Previously, using blood microarray data, our group demonstrated that transcriptomic profiling was able to modestly discriminate AOA2 patients from controls with a sensitivity of 67% and a specificity of 85% (Fogel et al. 2014). Although this result suggested that it would be possible to develop a disease biomarker for AOA2, this analysis was previously beyond our capabilities due to low resolution from microarray and small cohort size. Using RNA sequencing and WGCNA, here we identify a specific transcriptional network, module R22, that was able to more effectively classify patients from our training group with 73% sensitivity and 97% specificity. In the combined cohort, module R22 only showed a sensitivity of 53% in individuals and 70% in families as having AOA2. While the sensitivity could be further maximized by expanding the transcriptomic signature to be more permissive, the tradeoff was a loss of specificity (as low as 80%). In an independent derivation of the biomarker for AOA2, the Pigengene tool identified a two-module model, ME20 and ME31 (equivalent to modules R22 and R5 in the initial derivation), that had the best overall classification for the training cohort with a 100% sensitivity and 97% specificity, a dramatic improvement compared to our previous study and an increase of 27% sensitivity over the single-module model. Correspondingly, the two-module model identified 72% of individuals and 80% of families with AOA2 in the combined cohort. In comparison to module R22, the two-module ME20 and ME31 model identified 5 patients from 3 families that module R22 did not while module R22 identified 1 patient from a family that the two-module combination did not. In 1 family, both methods identified different individuals from within the same family. Additionally, the two-module combination identified 3 additional patients from 2 families versus module R22. Overall, the two-module model was able to classify seven additional patients and two additional families when compared to the single-module model.

We observed concordance between AOA2 disease-associated expression profiles across different tissues (human blood and fibroblast), across species (human tissue and mouse cerebellum), and across technology platforms (microarray and RNA sequencing) (Fig. S2) (Fogel et al. 2014), suggesting the potential for broad utility of transcriptional biomarkers. However, the most significant network, module R22, only overlapped with a single module in blood (B14) but not with any of the other modules from cultured human fibroblasts or mouse cerebellum (Fig. S2), suggesting that it may be tissue specific. This may explain why module R22 was the most discriminating module in patient blood. Notably, the R22 biomarker showed 100% sensitivity and 100% specificity in differentiating AOA2 from other neurological diseases with clinical overlap (Breen et al. 2023; McGrath-Morrow et al. 2020; Napierala et al. 2017). Module R22 was found to be preserved in the ALS4 and PMS datasets, indicating that it is not a unique transcriptional profile to AOA2, however it was only disease-associated in AOA2 and not the other datasets. The preservation of R22 in the ALS4 and PMS datasets is therefore not surprising as both are derived from patient blood. However R22 is specific to loss of SETX function as the module was not disease-associated in PMS or even in ALS4, despite this being a gain-of-function disorder also due to mutation of *SETX*, consistent with previous observations with in vitro cell models (Fogel et al. 2014). Interestingly, we did observe an overlap between the disease-associated ALS4 module ALS4HsModule77 and R05 (*p* = 8.16E-07), which is part of the two-module biomarker model, emphasizing a close relationship does exist between these two related disorders AOA2 and ALS4, as both are due to mutation of *SETX*. Although not preserved, a module with modest similarity to R22 was noted in A-T (A-T32, *p* = 3.58E-05), suggesting module R22 may have been lost due to transcriptional effects of the underlying disorder. Further investigation of the genes present in R22 and those shared across these key modules would be valuable and could provide insight into the underlying biological processes or pathways involved in disease pathogenesis. Collectively, this suggests that R22 represents a functional aspect of SETX that is disrupted by the loss-of-function mutations causative in AOA2 and further supports its utility as a biomarker.

Both the single and two-module models performed inconsistently in classifying the most clinically definite patients from the replication cohort. Module R22 was able to identify only 9 out of 21 patients (43%) and 8 out of 13 families (62%) in the cross-validation replication group. Of these patients identified as AOA2 from this group, the biomarker identified more patients designated as clinically probable (6/9, 67%) than clinically definite patients (3/12, 25%). Similarly, the two-module model (ME20 and ME31) identified all clinically probable patients (9/9, 100%) and the same number of clinically definite patients as R22 (3/12, 25%). There are multiple potential reasons why this may have happened. One potential explanation may be due to the small size of the cohort. Due to the heterogeneity of the AOA2-associated transcriptional profile, samples used in the training set may not reflect the same expression profile as all the patients in the replication set. One key aspect is that a variety of variant types lead to AOA2 ranging from missense, nonsense, frameshift, and deletions (Table 1, Supplemental Table S1). Variants like nonsense, multi-exon deletions, and frameshifts generally cause complete loss of function but missense mutations could result in partial loss of function and retain some protein activity. Depending on the location of such mutations, the transcriptional profile could be affected more strongly or weakly. Our small cohort size may limit detection to only the most robust signals. A second explanation for incomplete classification may be that some of the patients who tested negative do not, in fact, have AOA2, which, pending the identification of an alternate etiology, cannot presently be refuted by any established method. Not all patients had comprehensive genetic evaluations (see Methods). However, this is unlikely to be the sole explanation as illustrated, for example, by patient AOA2-10 (Table 1, Supplemental Table S1) who has two loss-of-function variants and tested negative by both models of the transcriptional signature. Positioning of the mutations harbored by this patient suggests the SETX DNA/RNA helicase domain would have been truncated in both instances with severe loss-of-function consequences. Therefore, we cannot rule out a third possibility that there may be other factors contributing to modification of expression profiles in patients that tested negative including diet, medications, or other genes causing background genetic effects on transcription. Given the small size of the cohort, it was not possible to precisely match the training and replication cohorts on the basis of age, sex, race, ethnicity, and/or disease severity which may all contribute to transcriptional alterations. In one study, differences between transcriptomic age and chronological age were found to be associated with biological features linked to aging, such as blood pressure, cholesterol levels, fasting glucose, and body mass index (Peters et al. 2015). Close inspection of the expression profiles from the patients that were not identified as AOA2 showed profiles similar to control patient expression profiles but trending toward AOA2 subject profiles (Fig. 2A). Transcription is dynamic, longitudinal assessments were not performed, and blood may not always perfectly represent the status of the nervous system for milder cases. Supporting this, both the single-module and the two-module models were able to identify all patients that reported earliest age of onset within the first decade (4/4 patients, Table 1, Supplemental Table S1), which may reflect a more severe underlying molecular pathology. Ultimately, a much larger cohort may be necessary to develop a more comprehensive expression pattern. This is supported by the two-module model performing better than the single module model but more inclusive models failing due to oversampling bias. Unfortunately, AOA2 is a rare disorder. Although, AOA2 is thought to be the fourth most common recessive cerebellar ataxia with an estimated prevalence as high as 1 in 900,000 (Anheim et al. 2010) in one study, based on multiple genomic sequencing studies, the true prevalence is likely much lower.

We subsequently utilized this transcriptomic biomarker to verify the first likely pathogenic AOA2 mutation in a non-canonical *SETX* transcript. The c.7287 + 204 G > A variant would have been missed in routine genetic testing analysis. This finding may reflect pathological consequences of AOA2 as this shorter isoform is expressed in more brain regions and expressed at a higher rate in testis than the canonical transcript. Of note, male Setx^−^/^−^ mice have defective spermatogenesis and this phenotype has recently been observed in male AOA2 patients as well (Becherel et al. 2019). As this isoform is expressed in testis, it may be related to this reproductive phenotype. However, we cannot rule out this variant as contributing to pathogenesis via the canonical transcript, in whole or in part, based on our current study. Nevertheless, this discovery of a likely pathogenic coding variant in an alternative isoform expands the spectrum of mutations that contribute to AOA2 and other SETX related disorders. Potentially other similar mutations await discovery, as illustrated by the number patients with only a single pathogenic variant who tested positive on the biomarker assay (Table 1, Supplemental Table S1).

## Conclusion

The molecular signature we have developed here showed an overall 72% sensitivity, and 97% specificity as a disease biomarker and was able to uncover a novel gene mutation in a non-canonical *SETX* transcript. Our study shows that for genes expressed in accessible tissues, transcriptional biomarkers may provide a new means to validate sequence changes of uncertain significance as pathogenic for known genetic diseases. An obvious future application for such a transcriptional biomarker would be in clinical trials research where expression signatures may act to provide real-time estimation of disease activity, progression, and/or severity for disease-modifying treatments and gene therapies.

## Supplementary Information

Supplementary Material 1: Supplemental Figure S1. Reanalysis of published AOA2 microarray data and correlation with disease-associated modules. A. human blood, B. patient fibroblast, C. Setx-/- mouse cerebellum.

Supplementary Material 2: Supplemental Figure S2. The AOA2 transcriptional biomarker signature is conserved across tissues, species, technology platforms, and is disease-specific. R = AOA2 patient blood RNA. B = AOA2 patient blood RNA. F = AOA2 patient fibroblast RNA. M = Setx-/- mouse cerebellum RNA. ALS4 = ALS4 patient blood RNA. The R and ALS4 datasets are derived from RNA sequencing while the others are microarray analysis.

Supplementary Material 3: Supplemental Figure S3. Validation of the R22 classifier in other neurodegenerative disease datasets. A. Comparison of module R22 preservation in other neurodegenerative datasets. B. Comparison of other neurodegenerative disease datasets preserved in the R module dataset. ALS4 = Amyotrophic lateral sclerosis type 4, A-T = Ataxia telangiectasia, FRDA = Friedreich’s ataxia, PMS = Phlean-McDermic syndrome.

Supplementary Material 4: Supplemental Table S1. Detailed Characteristics of the 20 families included in this study. Supplemental Table S2. Blood RNA-seq Module Membership. Supplemental Table S3. Module R05 Gene Ontology. Supplemental Table S4. Module R10 Gene Ontology. Supplemental Table S5. Module R20 Gene Ontology. Supplemental Table S6. Module R22 Gene Ontology. Supplemental Table S7. Module R23 Gene Ontology. Supplemental Table S8. AOA2 Transcriptional Biomarker Validation. Supplemental Table S9. Differentially Expressed Genes and Gene Ontology.

## Data Availability

The data generated in this manuscript are available from Gene Expression Omnibus (GEO). Published expression data presented in this manuscript are derived from the following studies in GEO: GSE61400 and GSE142842. Published sequence data presented in this manuscript are derived from the following accessions from the Sequence Read Archive (SRA): SRP394132 and SRP118922.

## Abbreviations

ACMG: American College of Medical Genetics and Genomics
AFP: Alpha-fetoprotein
ALS4: Amyotrophic lateral sclerosis type 4
AOA1: Ataxia with oculomotor apraxia type 1
AOA2: Ataxia with oculomotor apraxia type 2
AOA4: Ataxia with oculomotor apraxia type 4
A-T: Ataxia-telangiectasia
ATLD1: Ataxia-telangiectasia-like disorder 1
ATLD2: Ataxia-telangiectasia-like disorder 2
CADD: Combined Annotation Dependent Depletion
CQN: Conditional Quantile Normalization
FPKM: Fragments Per Kilobase of transcript per Million mapped reads
FRDA: Friedreich ataxia
GO: Gene ontology
HGMD: Human Gene Mutation Database
ME: Module Eigengene
PMS: Phelan-McDermid syndrome
SCAR26: Spinocerebellar ataxia, autosomal recessive 26
SETX: Senataxin
SNVs: Single nucleotide variants
VUS: Variants of uncertain clinical significance
WGCNA: Weighted Gene Co-expression Network Analysis
