# Supplementary material for: Developing a disease-specific accessible transcriptional signature as a biomarker for ataxia with oculomotor apraxia type 2
Source: Mol Med. 2025 May 24;31:205. doi: 10.1186/s10020-025-01257-8 (PMC12103034; doi:10.1186/s10020-025-01257-8)
Supplement: Supplementary file 1 — Supplementary Material 1: Supplemental Figure S1. Reanalysis of published AOA2 microarray data and correlation with disease-associated modules. A. human blood, B. patient fibroblast, C. Setx-/- mouse cerebellum. [file 10020_2025_1257_MOESM1_ESM.pptx]

## Slide 1
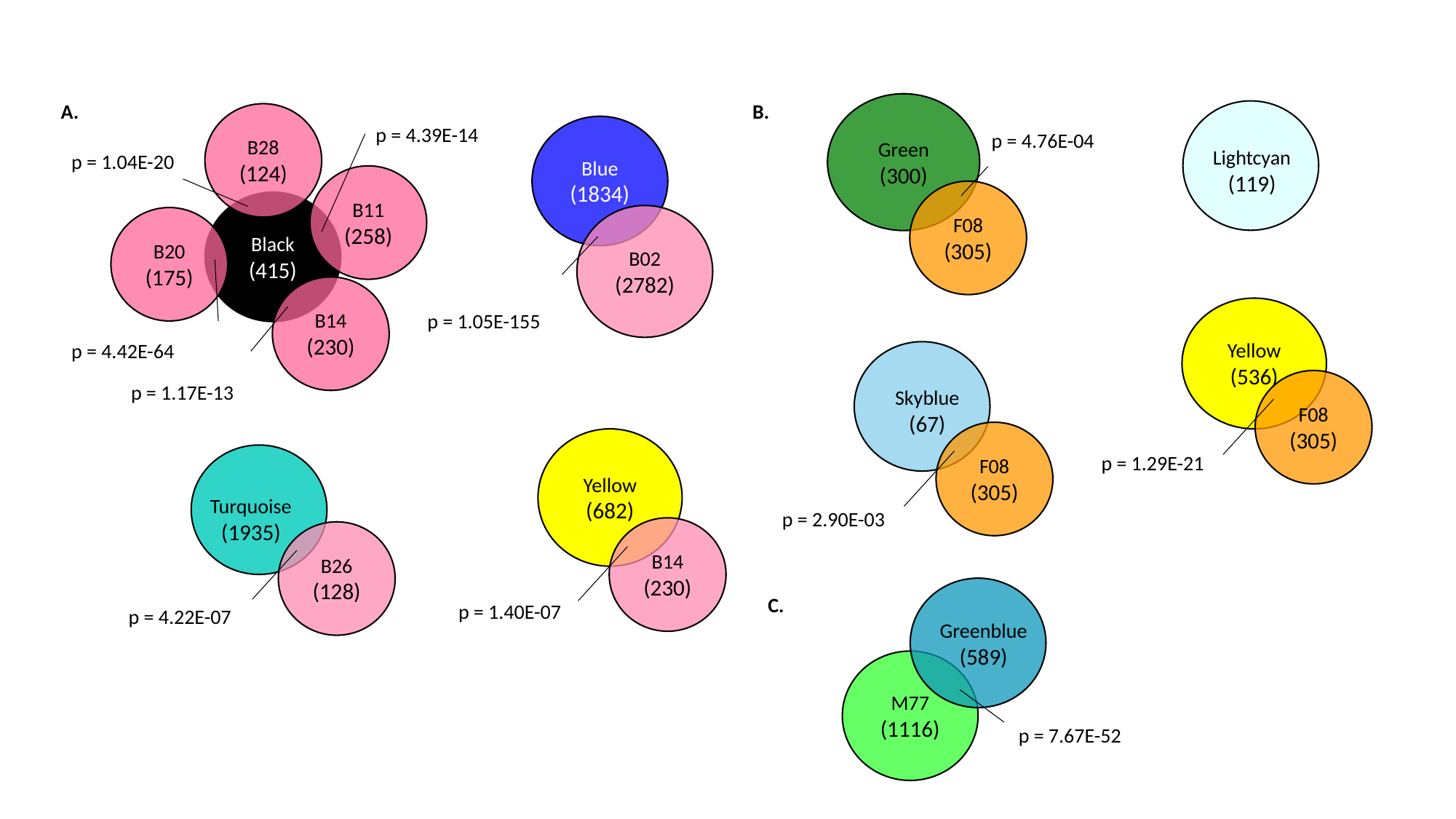

A.
B.
Green
(300)
p = 4.76E-04
F08
(305)
Lightcyan
(119)
B28
(124)
p = 4.39E-14
p = 1.04E-20
B11
(258)
Black
(415)
B20
(175)
B14
(230)
p = 4.42E-64
p = 1.17E-13
Blue
(1834)
B02
(2782)
p = 1.05E-155
Yellow
(536)
F08
(305)
p = 1.29E-21
Skyblue
(67)
F08
(305)
p = 2.90E-03
Yellow
(682)
B14
(230)
p = 1.40E-07
Turquoise
(1935)
B26
(128)
p = 4.22E-07
Greenblue
(589)
M77
(1116)
p = 7.67E-52
C.
