# Supplementary figures and images for: Developing a disease-specific accessible transcriptional signature as a biomarker for ataxia with oculomotor apraxia type 2
Source: Mol Med. 2025 May 24;31:205. doi: 10.1186/s10020-025-01257-8 (PMC12103034; doi:10.1186/s10020-025-01257-8)

## Slide 1
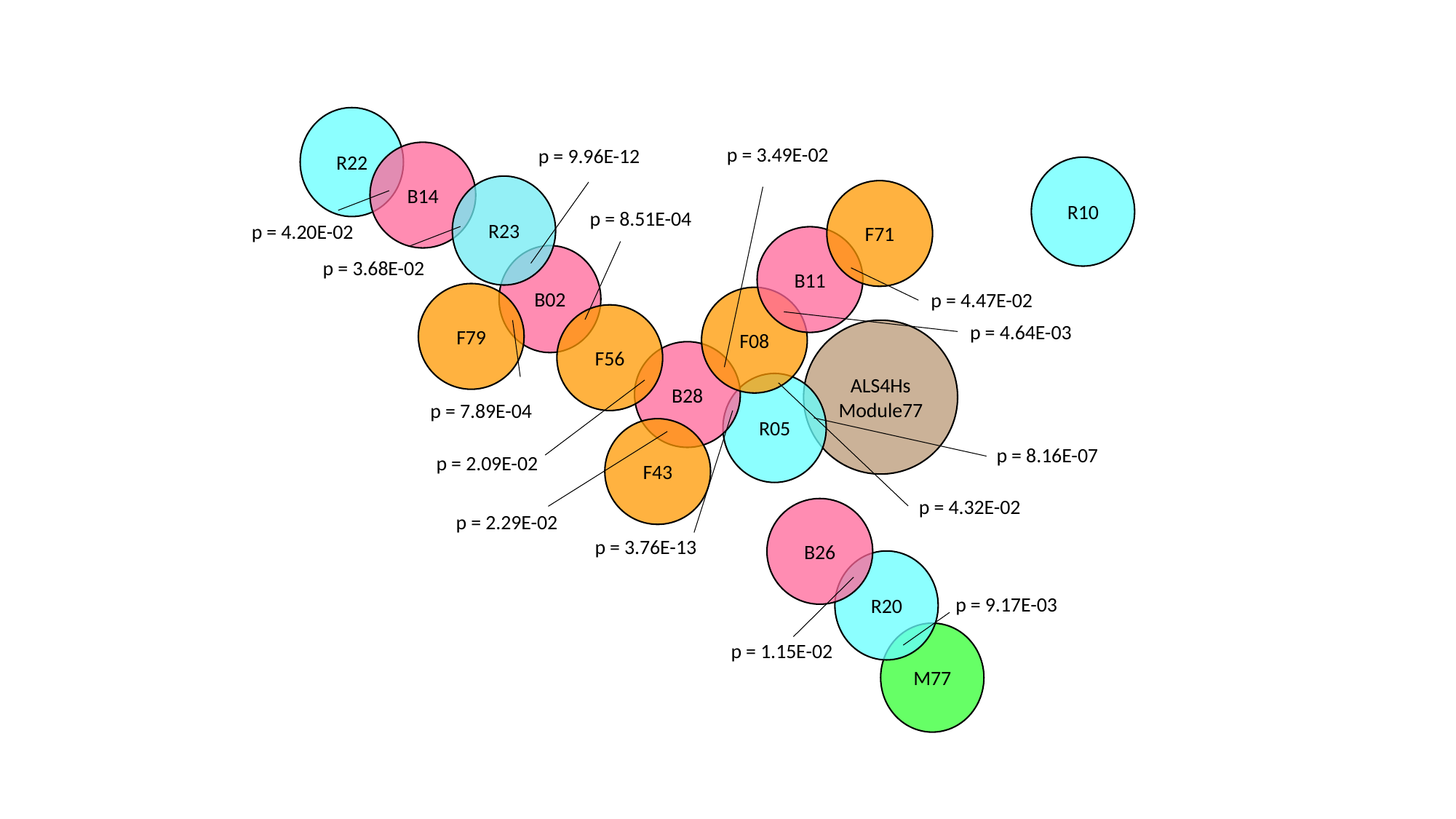

R22
p = 3.49E-02
p = 9.96E-12
B14
R10
R23
F71
p = 8.51E-04
p = 4.20E-02
B11
B02
p = 4.47E-02
F79
F08
F56
ALS4Hs
Module77
B28
R05
p = 7.89E-04
F43
p = 8.16E-07
p = 2.09E-02
p = 4.32E-02
B26
p = 2.29E-02
p = 3.76E-13
R20
p = 9.17E-03
M77
p = 1.15E-02
p = 3.68E-02
p = 4.64E-03

Supplement: Supplementary file 2 — Supplementary Material 2: Supplemental Figure S2. The AOA2 transcriptional biomarker signature is conserved across tissues, species, technology platforms, and is disease-specific. R = AOA2 patient blood RNA. B = AOA2 patient blood RNA. F = AOA2 patient fibroblast RNA. M = Setx-/- mouse cerebellum RNA. ALS4 = ALS4 patient blood RNA. The R and ALS4 datasets are derived from RNA sequencing while the others are microarray analysis. [file 10020_2025_1257_MOESM2_ESM.pptx]
