# Supplementary material for: Developing a disease-specific accessible transcriptional signature as a biomarker for ataxia with oculomotor apraxia type 2
Source: Mol Med. 2025 May 24;31:205. doi: 10.1186/s10020-025-01257-8 (PMC12103034; doi:10.1186/s10020-025-01257-8)
Supplement: Supplementary file 3 — Supplementary Material 3: Supplemental Figure S3. Validation of the R22 classifier in other neurodegenerative disease datasets. A. Comparison of module R22 preservation in other neurodegenerative datasets. B. Comparison of other neurodegenerative disease datasets preserved in the R module dataset. ALS4 = Amyotrophic lateral sclerosis type 4, A-T = Ataxia telangiectasia, FRDA = Friedreich’s ataxia, PMS = Phlean-McDermic syndrome. [file 10020_2025_1257_MOESM3_ESM.pptx]

## Slide 1
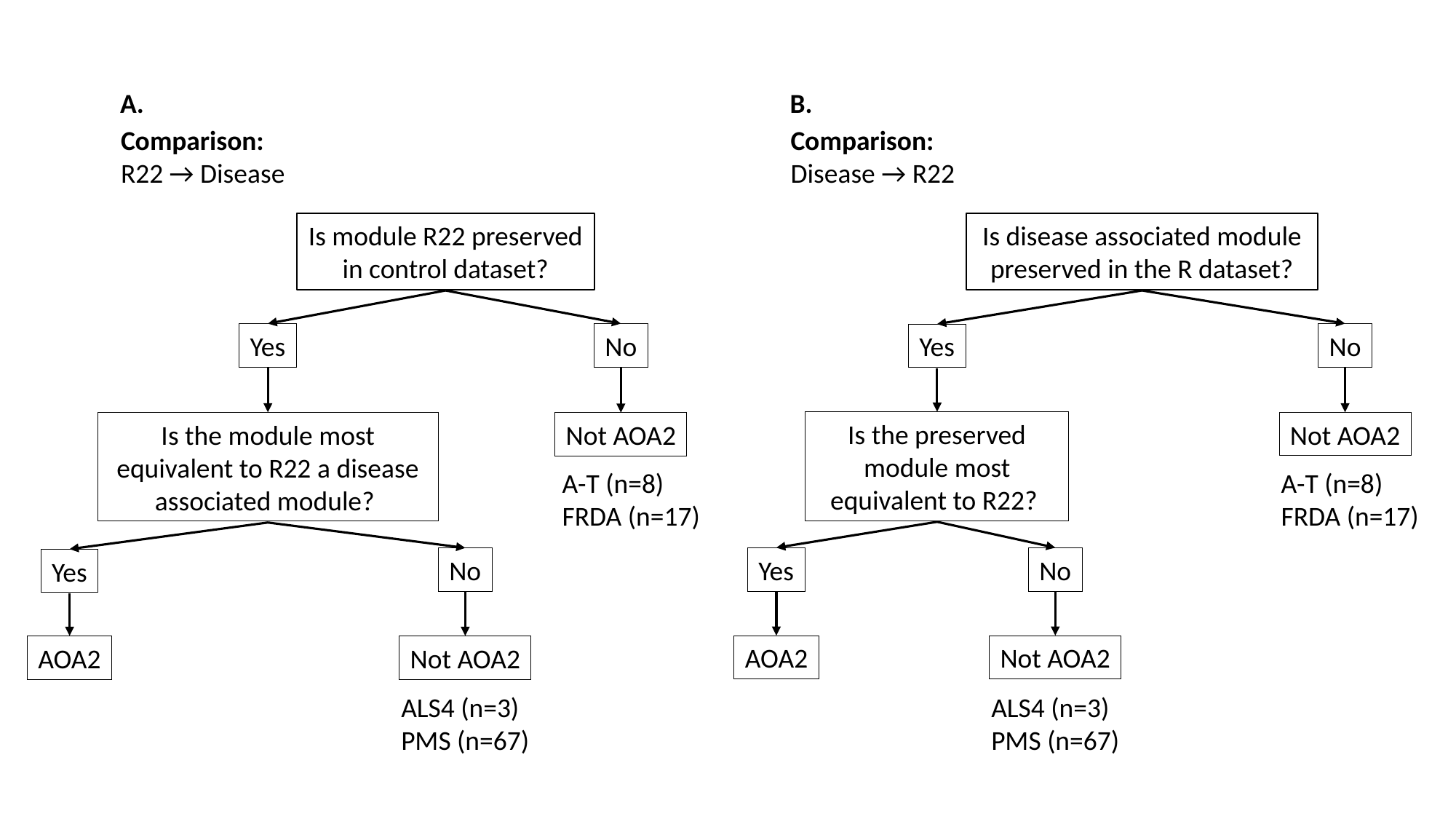

A.
B.
Comparison:
R22 → Disease
Comparison:
Disease → R22
Is module R22 preserved in control dataset?
Yes
No
Is the module most equivalent to R22 a disease associated module?
Not AOA2
No
Yes
AOA2
Not AOA2
A-T (n=8)
FRDA (n=17)
ALS4 (n=3)
PMS (n=67)
Is disease associated module preserved in the R dataset?
No
Yes
Is the preserved module most equivalent to R22?
Not AOA2
Yes
No
AOA2
Not AOA2
A-T (n=8)
FRDA (n=17)
ALS4 (n=3)
PMS (n=67)
